# Integrated Transcriptomic and Metabolomic Profiling Reveals the Involvement of the miR397-5p–SbLAC14 Module in Condensed Tannin Accumulation in Developing Sorghum Seeds

**DOI:** 10.3390/plants15152339

**Published:** 2026-07-29

**Authors:** Yannan Shi, Yongchao Guo, Jinping Wang, Zhifang Wang, Zhiyin Jiao, Xue Ma, Shilong Li, Baoqing Dun, Haifang Sun, Jingtian Niu, Peng Lv, Guoquan Liu

**Affiliations:** 1 Institute of Millet Crops, Hebei Academy of Agriculture & Forestry Sciences, Hebei Branch of China National Sorghum Improvement Center, Shijiazhuang 050035, China; 18251912885@163.com (Y.S.); gycxhxbc115@163.com (Y.G.); wangjinping@haafs.org (J.W.); wangzhifang@haafs.org (Z.W.); zhiyin_jiao@163.com (Z.J.); maxue199@163.com (X.M.); njt108545810@126.com (J.N.); 2 China-Australia Joint Laboratory for Sorghum Transformation and Genome Editing, Shijiazhuang 050035, China; 3 Hebei Key Laboratory of Crop Stress Biology, College of Agronomy and Biotechnology, Hebei Normal University of Science and Technology, Qinhuangdao 066000, China; 13561755650@163.com; 4 Zhongyuan Research Center, Chinese Academy of Agricultural Sciences, Xinxiang 453000, China; dunbaoqing@caas.cn; 5 Institute of Agricultural Information and Economy, Hebei Academy of Agriculture and Forestry Sciences, Shijiazhuang 050051, China; sunhaifang@haafs.org; 6 Centre for Crop Science, Queensland Alliance for Agriculture and Food Innovation, The University of Queensland, St. Lucia, QLD 4072, Australia

**Keywords:** sorghum, miR397, condensed tannin, Laccase14, seed color

## Abstract

Sorghum seeds accumulate substantial amounts of condensed tannins (CTs), which are also referred to as proanthocyanidins (PAs), contributing to their characteristic astringent taste. Flavan-3-ol polymers, known as PAs, are sequestered within plant vacuoles and become catalytically activated via laccase enzymes. However, the biological roles and regulatory pathways of laccases in sorghum are still largely unclear. Here, integrated transcriptomic and metabolomic profiling of developing sorghum seeds identified 7942 differentially expressed genes between low- and high-CT lines, with Kyoto Encyclopedia of Genes and Genomes (KEGG) enrichment revealing flavonoid biosynthesis as a key pathway; weighted gene co-expression network analysis (WGCNA) further pinpointed *SbLAC14* as a hub gene within the module most strongly correlated with CT content. We then examined its regulation by microRNA397 (SbmiR397-5p). Dual-luciferase assays confirmed the binding of SbmiR397-5p to SbLAC14 in co-transformed tobacco leaves. Overexpressing *SbLAC14* in transgenic *Arabidopsis* significantly increased CT accumulation while decreasing catechin and epicatechin levels. Furthermore, transgenic plants overexpressing miR397 (OEmiR397-5p) exhibited reduced CT content, accompanied by a lightening of seed color. Conversely, transgenic lines overexpressing a miR397-insensitive laccase transcript exhibited a reversed phenotypic outcome. Our findings indicate that SbmiR397-5p negatively regulates the expression of *SbLAC14* in relation to CT biosynthesis, identifying it as a potential target for manipulating CT metabolism in sorghum. Those results provide a genetic entry point for metabolic engineering and breeding efforts aimed at modulating grain phenolic profiles.

## 1. Introduction

Sorghum [*Sorghum bicolor* (L.) Moench] is a significant cereal grass, acting as a primary food source for more than 500 million individuals across over 30 tropical and subtropical nations [1]. Due to its remarkable resilience to drought, high temperatures, and low-fertility soils, sorghum is essential for maintaining food security in the context of shifting climatic conditions [2]. Beyond its importance as a staple food, sorghum is widely used for animal feed, bioethanol production, and the brewing of traditional alcoholic beverages, particularly in Asia and Africa. In China, sorghum grain serves as a key ingredient for making Baijiu, a conventional distilled spirit, where grain quality traits such as seed color, tannin content, and polyphenol composition directly influence flavor and processing characteristics [3]. These diverse applications underscore the agronomic and economic value of sorghum and highlight the importance of understanding the molecular mechanisms regulating seed quality traits, including condensed tannin biosynthesis. The tannins present in sorghum grains are important criteria for industrial processing [4]. However, investigations into tannin biosynthesis within sorghum grains remain limited. Elucidating the regulatory mechanisms of tannin accumulation in sorghum seeds will facilitate the genetic improvement in grain quality traits for diverse end-uses, including feed and brewing applications.

Tannins are a class of polyphenolic secondary compounds possessing molecular masses between 500 and 3000, and they are commonly found across the plant kingdom [5]. According to their chemical structural characteristics, tannins are typically divided into two principal groups: hydrolyzable tannins and condensed tannins [6,7,8]. Hydrolyzable tannins possess complex structures composed of various components, primarily derived from oak trees [9]. Condensed tannins are commonly found across the plant kingdom, including gymnosperms and angiosperms, with a higher prevalence in dicotyledons than in monocotyledons [10]. Tannins are distributed at varying concentrations among different plant tissues, such as seeds, roots, stems, leaves, buds, pods, and fruits [11]. Tannins have traditionally been regarded as “anti-nutritional factors” due to their potential adverse effects on human and animal nutrition [12,13]. However, recent research has highlighted their important role in brewing quality. In addition, tannins exhibit functions such as metal ion chelation, protein precipitation, and biological antioxidant activity, leading to their widespread applications in food, pharmaceuticals, cosmetics, papermaking, and wastewater treatment [4,14].

The tannins in sorghum grains are primarily condensed tannins, with a smaller proportion of hydrolyzable tannins. Condensed tannins, also known as proanthocyanidins (PAs), are synthesized through a branch of the flavonoid biosynthetic pathway [15]. The biosynthetic process is initiated by chalcone synthase (CHS), which catalyzes the formation of chalcone from phenylpropanoid precursors. Chalcone undergoes isomerization via chalcone isomerase (CHI), yielding the flavanone naringenin, which is then transformed into dihydroflavonols by subsequent enzymatic reactions. The reduction of dihydroflavonols into colorless leucoanthocyanidins is catalyzed by dihydroflavonol reductase (DFR), which are further oxidized by anthocyanidin synthase (ANS) to form colored anthocyanidins [16]. The colorless anthocyanin precursor (leucoanthocyanidin) and anthocyanin precursor are subsequently converted to flavan-3-ol by leucoanthocyanin reductase (LAR) and anthocyanin reductase (ANR), respectively, which are then polymerized through largely unknown mechanisms to form proanthocyanidins [17].

Plant laccases (LACs) belong to the blue copper oxidase group, also known as the p-diphenol:dioxygen oxidoreductase family, and were commonly present across various higher plant species and have been implicated in PA biosynthesis [18]. The laccase gene *DkLAC2* has been proposed to be involved in PA biosynthesis in persimmon, similar to the proposed role of *SmLAC1* in *Salvia miltiorrhiza* and *Populus trichocarpa* [19,20]. Both *DkLAC2* and *SmLAC1* catalyze the polymerization of flavan-3-ol to produce PA and are regulated by microRNA397.

Research on the molecular basis of condensed tannins (CTs, sorghum proanthocyanidins) synthesis and regulation in sorghum remains at an early stage. Wu et al. cloned the homologous gene *Tannin1* (*Tan1*, Sobic.004G280800) from *Arabidopsis* using a genetic linkage map [1]. This gene produces a WD40 repeat-containing protein which functions as part of the WD40-MYB-bHLH complex to regulate anthocyanin and PA biosynthesis. In 2019, the same team utilized GWAS technology to clone *Tannin2* (*Tan2*, Sobic.002G076600), which encodes a bHLH transcription factor that similarly participates in anthocyanin and CT biosynthesis [13]. Apart from *Tannin1* and *Tannin2*, other genes related to tannin synthesis have yet to be identified. In this study, we employed a combined multi-omics strategy to uncover the regulatory circuitry that controls condensed tannin metabolism throughout sorghum seed maturation. Integrated metabolomic and transcriptomic profiling uncovered stage-dependent expression profiles of *SbLAC* genes as well as their putative transcriptional regulators. Bioinformatic analyses combined with experimental validation demonstrated that miR397 targets *SbLAC14*, promoting its transcript degradation and thereby post-transcriptionally regulating proanthocyanidin biosynthesis. These results deepen our knowledge of CT biosynthesis in sorghum seeds and offer important guidance for improving grain quality, increasing nutritional value, and broadening its industrial applicability across multiple sectors.

## 2. Results

### 2.1. Dynamic Accumulation of CTs During Sorghum Seed Development

**Figure 1:**
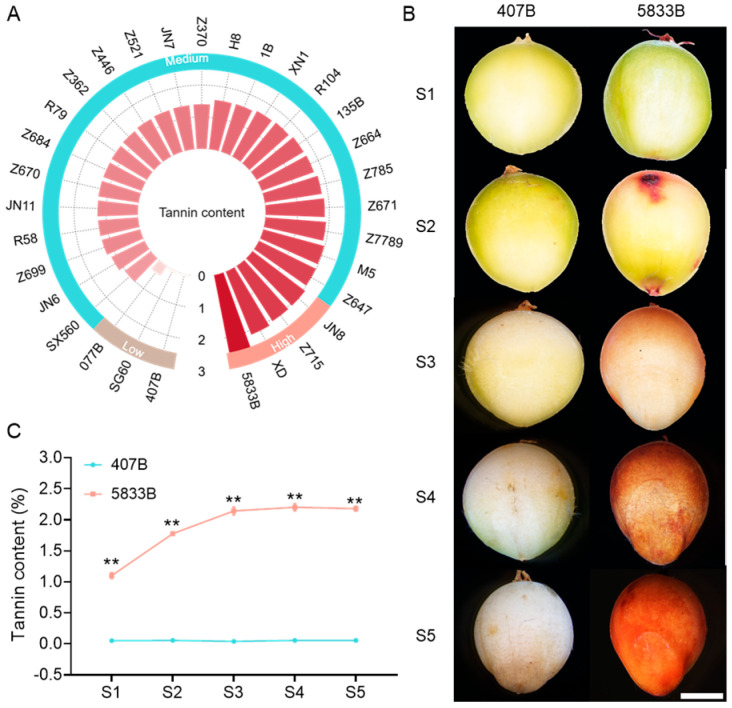
Analysis of CT content diversity and its developmental dynamics in sorghum seeds. (**A**) Distribution of CT content (%) in 32 sorghum germplasms. The accessions were categorized into three groups: low (≤0.5%), medium (1–2%), and high (>2%) CT content. Values shown are mean ± SD from three biological replicates (n = 3). (**B**) Phenotypic evolution of seeds from lines 407B (low-CT) and 5833B (high-CT) across five successive developmental stages (S1 to S5, representing increasing maturity). The distinct brown pigmentation associated with CT accumulation is visibly apparent in 5833B from stage S3 onward. Scale bars: 2 mm. (**C**) Quantitative changes in seed tannin content in lines 407B and 5833B throughout the developmental stages S1 to S5. Data points represent the mean ± SD (n = 3, ** *p* < 0.01; Student’s *t*-test). Note the steep increase in CT accumulation in 5833B after stage S2, contrasting with the consistently low levels in 407B.

To investigate condensed tannin (CT) biosynthesis, 32 sorghum lines were analyzed for CT content (Figure 1A). CT levels ranged from 0.08% to 2.5%, with the lowest observed in 407B (0.08%) and the highest monitored in 5833B (2.5%). These two genotypes, exhibiting contrasting CT contents, were selected for further study. Stereomicroscopic observation of 407B and 5833B grains at 5, 10, 15, 20, and 25 days post-anthesis (S1–S5) revealed distinct phenotypic changes, including grain size variation and progressive loss of green pigmentation (Figure 1B). This color transition was temporally associated with CT accumulation in the seed coat (Figure S1), although the underlying mechanism linking CT dynamics to pigment changes requires further investigation.

CT content was quantified across developmental stages (Figure 1C). In 5833B, CT levels increased from 1.10% (S1) to a peak of 2.20% (S4), while 407B showed minimal fluctuation (0.40–0.56%). At S5, CT content in both lines remained similar to S4. These dynamics indicate a net accumulation of CTs during early to mid-seed development, with levels stabilizing or reaching a plateau at later stages (Figure S1).

### 2.2. Transcriptional Changes Involved in Seed Coat Color Formation

**Figure 2:**
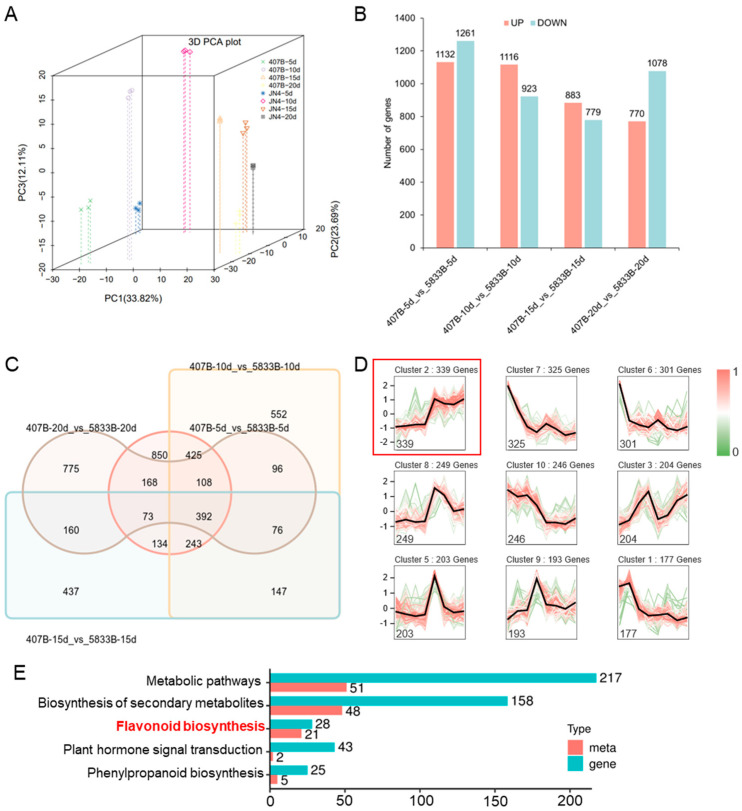
Transcriptomic analysis of developing seeds in 407B and 5833B. (**A**) PCA of transcriptome data from 407B and 5833B across four developmental stages (S1–S4). PC1, PC2, and PC3 explain 33.82%, 23.69%, and 12.11% of variance, respectively. (**B**) Number of DEGs (|log_2_FC| ≥ 1, adj.*p* < 0.05) between 407B and 5833B at each stage. Genes with increased (red) and decreased (blue) expression in 5833B are displayed. (**C**) Venn diagram showing overlap of DEGs across S1–S4. (**D**) K-means clustering of all DEGs. The x-axis represents the eight samples arranged by developmental stage (S1–S4, left to right), with the two varieties 407B and 5833B shown at each stage. The y-axis indicates relative expression levels (z-scored FPKM values). Nine clusters were identified based on expression patterns. Cluster 2 (highlighted by red frame) shows specific up-regulation in 5833B at S1–S4. (**E**) Top 5 enriched KEGG pathways for Cluster 2 genes.

To investigate the transcriptional regulatory mechanisms underlying seed color formation in sorghum, we performed RNA sequencing (RNA-seq) on seeds at four developmental stages (S1–S4). The high correlation (R^2^ > 0.8) between RNA-seq data and qRT-PCR validation across seed developmental stages (Figure S2) confirms the reliability and biological relevance of the transcriptomic profiles. Principal component analysis (PCA) revealed clear transcriptional segregation among various developmental stages, and biological replicates formed tight clusters, validating the strong reproducibility of the dataset (Figure 2A). Across all developmental stages, a total of 7942 DEGs were detected. Specifically, S1 (407B-5d_vs_5833B-5d), S2 (407B-10d_vs_5833B-10d), S3 (407B-15d_vs_5833B-15d), and S4 (407B-20d_vs_5833B-20d) contained 2393, 2039, 1662, and 1848 DEGs, respectively (Table S1; Figure 2B). To visualize the overlap and specificity of transcriptional changes across different comparisons, we constructed a Venn diagram (Figure 2C). The diagram showed that 392 DEGs were common to all comparisons, whereas 850, 552, 437, and 775 DEGs were unique to the comparison between the two genotypes at the S1–S4 stage, respectively. These results indicate that a substantial portion of the transcriptomic response is genotype- and stage-specific.

To explore temporal expression dynamics, DEGs were grouped into nine clusters via K-means clustering based on their expression profiles (Table S2; Figure 2D). Cluster 2 comprised genes upregulated in 5833B relative to 407B. KEGG enrichment analysis performed on this cluster showed a marked enrichment of pathways involved in flavonoid biosynthesis, plant hormone signal transduction, and phenylpropanoid biosynthesis (Figure 2E) suggesting a potential role for these genes in pigment accumulation.

### 2.3. Analysis of CTs and Anthocyanins Biosynthesis During Sorghum Seed Ripening

**Figure 3:**
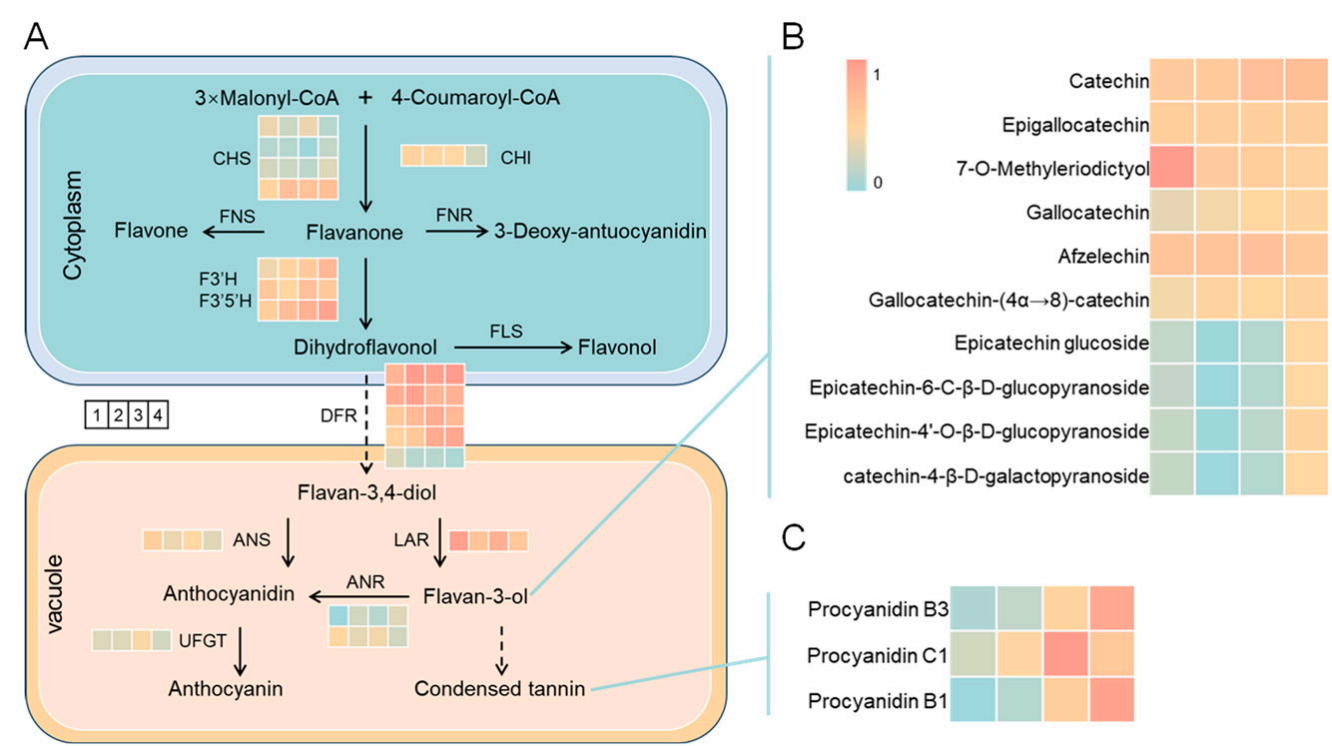
Expression of tannin pathway genes and accumulation of flavan-3-ols in 407B and 5833B. (**A**) Heatmap of anthocyanin and condensed tannin biosynthesis gene expression. Log_2_-transformed fold change values were normalized across triplicates (scale 0–1). CHS, chalcone synthase; CHI, chalcone isomerase; F3′H, ffavonoid 3′-hydroxylase; F3′5′H, ffavonoid 3′5′-hydroxylase; FLS, ffavonol synthase; DFR, dihydrof lavonol 4-reductase; ANS, anthocyanidin synthase; LAR, Leucoanthocyanidin reductase; UFGT, UDP-glucose:anthocyanidin 3-O-glucosyltransferase; ANR, Anthocyanidin Reductase. (**B**) Heatmap of flavan-3-ols (condensed tannin precursors). Metabolite levels were log_2_-transformed and normalized across triplicates (scale 0–1). (**C**) Heatmap of PAs (condensed tannin). Metabolite levels were log_2_-transformed and normalized across triplicates (scale 0–1). Numbers 1–4 indicate pairwise comparisons: 1, 5833B-5d vs 407B-5d; 2, 5833B-10d vs 407B-10d; 3, 5833B-15d vs 407B-15d; 4, 5833B-20d vs 407B-20d. Relative abundance is indicated by a color gradient ranging from blue (low) to red (high).

By combining metabolomic and transcriptomic profiling, we comprehensively characterized 19 core enzyme-encoding genes participating in the flavonoid and anthocyanin biosynthetic routes (Figure 3A; Table S3), alongside 13 representative differentially accumulated metabolites (DAMs) (Figure 3B,C; Table S4). In the upstream stages of these pathways, most members of the CHS, CHI, and F3’H gene families exhibited consistently high expression across all developmental stages (S1–S4), thus guaranteeing an ongoing provision of precursors for flavonoid production and laying a solid groundwork for downstream metabolic reactions.

During the middle phase of the pathway, increased expression of DFR genes promoted the buildup of flavan-3,4-diol. Enhanced activities of downstream LAR genes facilitated the conversion of flavan-3,4-diol into proanthocyanidin precursors (flavan-3-ols), promoting condensed tannin biosynthesis. In contrast, genes involved in anthocyanin biosynthesis, including ANS, ANR, and UFGT, were significantly downregulated, particularly in the later developmental stages (S3–S4).

These coordinated expression patterns reveal a metabolic flux redirection from anthocyanin production toward condensed tannin accumulation during sorghum seed maturation. The sustained activation of upstream flavonoid pathway genes, coupled with the differential regulation of branch-point enzymes (DFR/LAR vs. ANS/ANR/UFGT), preferentially channels metabolic intermediates into the proanthocyanidin biosynthetic route. This transcriptional reprogramming likely underpins the progressive accumulation of condensed tannins observed in pigmented sorghum genotypes such as 5833B, while limiting anthocyanin accumulation, ultimately shaping the distinct seed color and CT content phenotypes.

### 2.4. SbLACs Were Involved in CTs Biosynthesis of Sorghum Seed

**Figure 4:**
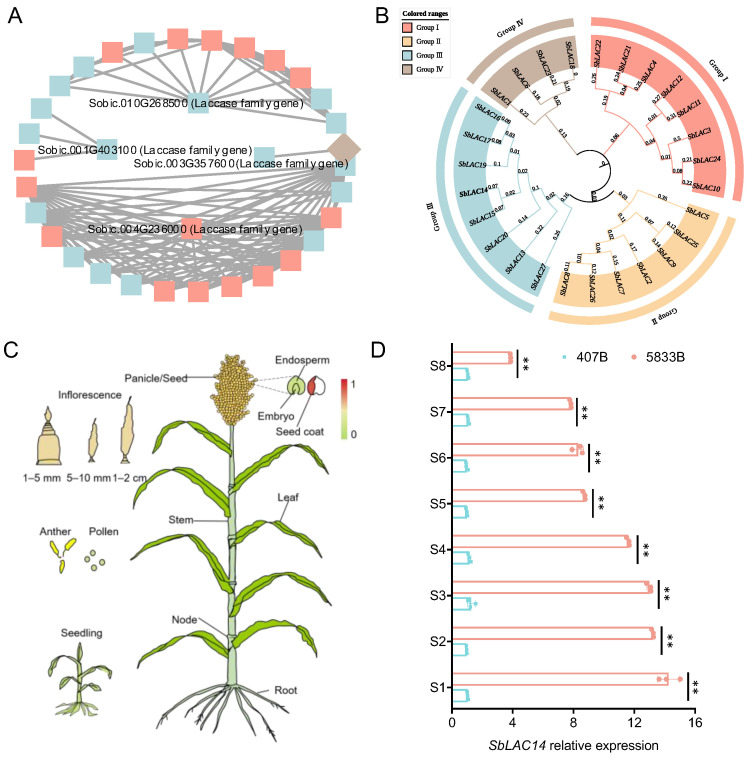
Identification and expression analysis of *SbLAC14* as a candidate gene associated with CT accumulation. (**A**) Using WGCNA, we identified co-expression gene modules correlated with distinct stages of sorghum seed development. (**B**) Phylogenetic analysis of the *LACCASE* gene family in sorghum. *SbLAC14* is highlighted in the tree. The tree was constructed using the neighbor-joining (NJ) method with MEGA 7.0, based on the amino acid sequences of LAC proteins from sorghum. Numbers above/below branches indicate bootstrap percentages (1000 replicates). Nodes with values 0 indicate bootstrap support <50%. (**C**) Tissue-specific expression pattern of *SbLAC14*. Transcript levels were examined in different tissues, showing predominant expression in the seed coat. (**D**) Expression dynamics of *SbLAC14* during seed development. Relative expression levels were measured at eight developmental stages (S1–S8), corresponding to 5, 10, 15, 20, 25, 30, 35, and 40 days after pollination (DAP). Values are mean ± SD from three replicates (n = 3, ** *p* < 0.01; Student’s *t*-test).

With the aim of uncovering critical DEGs associated with seed coat pigmentation throughout sorghum seed maturation, a metabolite–gene co-expression network was built via Weighted Gene Co-expression Network Analysis (WGCNA). This strategy enabled the detection of hub genes that may govern pigment deposition. A total of 18 co-expression modules were identified. Among these, the MEbrown module (containing 34 genes or proteins) showed the highest positive correlation with CT content (r = 0.9047, *p* = 3.43 × 10^−6^). This module was significantly enriched in genes involved in flavonoid/CT biosynthesis. Hub genes within this module (MM > 0.8, GS > 0.8) were further examined. Among the hub genes, we identified four laccase-encoding genes (Sobic.010G268500, Sobic.001G403100, Sobic.003G357600, and Sobic.004G236000 (Figure 4A). Previous research has established that laccases are well-known regulators of proanthocyanin biosynthesis in *Salvia miltiorrhiza* and *Populus trichocarpa* [19]. This suggests that these four laccases may play a critical role in the regulation of proanthocyanin in sorghum.

### 2.5. SbLAC14 Was Specifically Expressed During Sorghum Seed Coat Development

By performing genome-wide identification and phylogenetic analysis, we found that the sorghum laccase gene family consists of 27 members, which were classified into four distinct subfamilies (Figure 4B; Table S5). Subsequently, expression profiling by qRT-PCR of all 27 sorghum laccase genes across various tissues revealed that only *SbLAC14* displayed elevated expression in the seed coat (Figure 4C; Figure S3). To further investigate the temporal expression dynamics during seed development, qRT-PCR analysis was performed on *SbLAC14* using seed coat tissues collected at 5, 10, 15, 20, 25, 30, 35, and 40 days post-anthesis (DPA). Strikingly, *SbLAC14* exhibited consistently high transcript levels throughout all examined developmental stages (Figure 4D). This persistent and seed coat-specific expression strongly supports the conclusion that *SbLAC14* was specifically expressed during sorghum seed development, implicating its potential role in secondary cell wall thickening, pigmentation, or defense-related processes in the developing seed coat.

### 2.6. SbLAC14 Is a Candidate Gene Associated with the Polymerization of Flavan-3-ol to Form Condensed Tannins

To functionally characterize SbLAC14, we established a heterologous expression system in *Arabidopsis thaliana*. Transgenic lines overexpressing the *SbLAC14* coding sequence under the constitutive 35S promoter were generated. qRT-PCR analysis confirmed robust expression in two independent homozygous T3 lines (#OE-1 and #OE-2), with transcript levels 14.7-fold and 8.3-fold higher than wild-type (Col-0), respectively (Figure S4A), providing a suitable platform for functional assessment.

**Figure 5:**
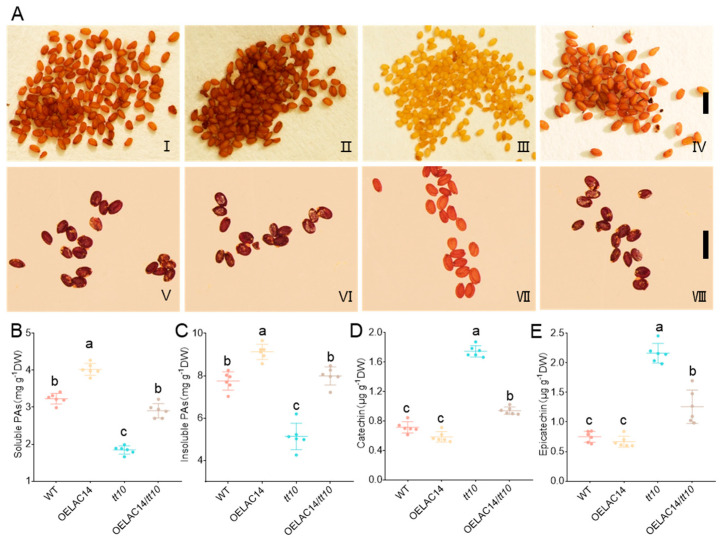
Functional complementation of the *Arabidopsis tt10* mutant by *SbLAC14*. (**A**) Seed phenotypes of four *Arabidopsis* lines: wild-type (WT, Col-0), SbLAC14-overexpressing WT (OE-LAC14/WT), *tt10* mutant, and *SbLAC14*-overexpressing *tt10* mutant (OE-LAC14/*tt10*). Panels I–IV show seeds stained without DMACA; panels V–VIII show seeds stained with DMACA, which specifically stains condensed tannins (blue coloration). Scale bars: 400 μm. (**B**–**E**) Quantification of soluble proanthocyanidins (PAs) (**B**), insoluble PAs (**C**), catechin (**D**), and epicatechin (**E**) levels in mature seeds of the four *Arabidopsis* lines. Values are shown as mean ± SD from three biological replicates (n = 3). Bars with different letters denote statistically significant differences at *p* < 0.05, as determined by one-way ANOVA followed by Tukey’s post hoc test.

The same 35S::*SbLAC14* construct was introduced into the *Arabidopsis tt10* mutant, which lacks functional *AtLAC15* and is defective in condensed tannin (CT) polymerization, resulting in a transparent seed coat phenotype with pale brown seeds (Figure 5A). Strikingly, seed color in *OELAC14*/*tt10* complemented lines was fully restored to the dark brown phenotype characteristic of wild-type seeds (Figure 5A). DMACA staining of developing seeds, which specifically detects CTs as blue precipitate, revealed that the strong blue signal absent in *tt10* mutants was completely reinstated in complementation lines, mirroring wild-type staining patterns (Figure 5A).

Quantitative biochemical analysis of mature seeds confirmed these observations. The *tt10* mutant accumulated significantly higher levels of flavan-3-ol monomers (catechin and epicatechin; ~2-fold increase over WT) with a corresponding ~50% reduction in polymeric CTs. Both complemented lines exhibited metabolite profiles indistinguishable from wild type, with monomer pools reduced to WT levels and CT content restored to 95–102% of WT concentrations (Figure 5B–E).

To examine the cellular distribution of *SbLAC14*, a recombinant SbLAC14-GFP fusion construct was expressed in rice protoplasts. The fusion protein localized predominantly to membrane-associated compartments (Figure S5B), in contrast to the diffuse cytosolic distribution observed in the GFP-only control. This membrane localization suggests SbLAC14 operates at a specific subcellular interface, potentially facilitating flavonoid substrate channeling or compartmentalization of the proanthocyanidin polymerization process.

Collectively, genetic complementation of the *tt10* phenotype, restoration of wild-type metabolite profiles, and membrane localization provide multilayered evidence that SbLAC14 functions as a membrane-associated laccase involved in catalyzing oxidative polymerization of flavan-3-ol monomers into condensed tannins during seed development.

### 2.7. miR397-5p Reduced CTs of Sorghum Seed Coat by Blocking SbLAC14 Expression

The miR397 family is highly conserved across plant species and is well documented to target laccase (LAC) genes [20,21]. According to the miRBase database (https://www.mirbase.org/hairpin/MI0010899, accessed on 20 March 2025), the sorghum genome contains only two miR397 family members (miR397-3p and miR397-5p). We therefore focused on validating the function of miR397-5p, the mature form of miR397-5p, in regulating SbLAC14.

**Figure 6:**
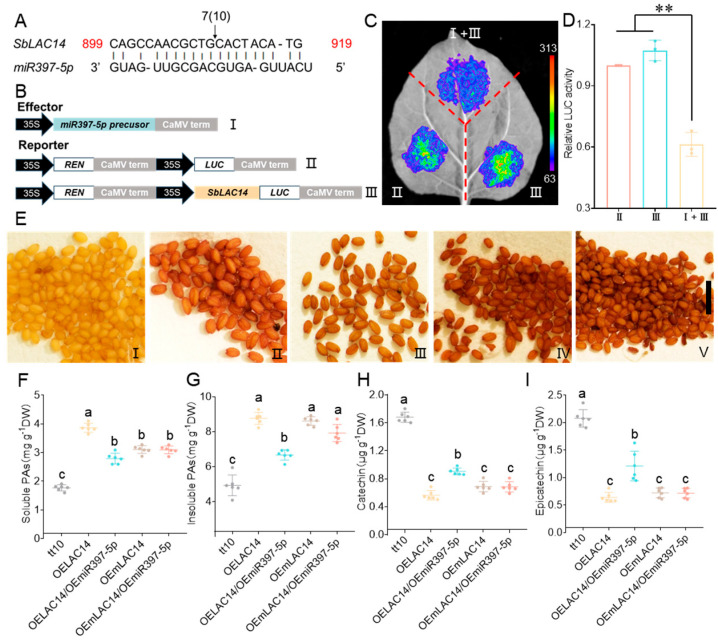
MiR397-5p targets *SbLAC14* and regulates condensed tannin accumulation in *Arabidopsis* seeds. (**A**) 5′ RACE analysis confirming the cleavage site of *SbLAC14* mRNA. The red numbers indicate nucleotide positions relative to the start codon; the black arrow marks the cleavage site. Seven out of ten sequenced clones mapped to this site. (**B**) Illustrations depicting the effector and reporter vectors employed in dual-luciferase reporter assays. Effectors included control, miR397-5p precursor, and mutated miR397-5p (mut-miR397-5p). Reporters contained the full-length *SbLAC14* sequence fused to the *LUC* gene. (**C**) Examples of luminescence signals observed in *Nicotiana benthamiana* leaves following co-infiltration with various effector and reporter combinations. (**D**) Quantitative analysis of relative *LUC* activity from (**C**). Values were normalized to the control group. Data are presented as mean ± SD (n = 3 biological replicates). Values are mean ± SD from three biological replicates (n = 3). Asterisks denote significant differences versus the control (** *p* < 0.01; Student’s *t*-test). (**E**) Seed phenotypes of five *Arabidopsis* lines: *tt10* mutant, *SbLAC14*-overexpressing *tt10* (OE-LAC14/*tt10*), OE-LAC14/OE-miR397-5p in *tt10* background, mutated *SbLAC14*-overexpressing *tt10* (OE-mLAC14/*tt10*), and OE-mLAC14/OE-miR397-5p in *tt10* background. Scale bars: 400 μm. (**F**–**I**) Quantification of soluble proanthocyanidins (PAs) (**F**), insoluble PAs (**G**), catechin (**H**), and epicatechin (**I**) levels in mature seeds of the five *Arabidopsis* lines. Values are shown as mean ± SD from three biological replicates (n = 3). Bars bearing distinct letters differ significantly at *p* < 0.05, as determined by one-way ANOVA with Tukey’s post hoc test.

Direct molecular targeting of *SbLAC14* by miR397-5p was confirmed through 5′ RACE analysis. Cleavage products of *SbLAC14* mRNA from developing sorghum seeds were isolated and sequenced, with the predominant cleavage site mapped to the region complementary to nucleotides 10–11 of miR397-5p (Figure 6A), confirming in vivo miRNA-directed cleavage. Dual-luciferase reporter assays in *Nicotiana benthamiana* provided further validation. Co-expression of miR397-5p with a reporter containing the wild-type *SbLAC14* 3′UTR reduced luciferase activity by ~60% (Figure 6B–D). This suppression was abolished when the miRNA-binding site was mutated, confirming interaction specificity.

To investigate post-transcriptional regulation, we generated transgenic *tt10 Arabidopsis* mutants overexpressing the sorghum miR397-5p precursor (*OEmiR397*). qRT-PCR confirmed that mature miR397-5p levels increased >8.2-fold in three independent homozygous lines (#OE-4, #OE-5, #OE-7) relative to wild-type (Figure S5C). Concurrently, *SbLAC14* transcript abundance in these lines was reduced by >80%, whereas mutated *LAC14* (*mLAC14*) transcript levels showed no significant decrease, confirming miRNA-mediated downregulation (Figure S5C). Phenotypic analysis revealed that OEmiR397 seeds exhibited lighter brown coloration compared to *OELAC14* seeds. However, this color difference was not observed between OEmiR397 and *OEmLAC14* seeds, indicating that the phenotype depends on an intact miR397-5p target site (Figure 6E). Metabolite quantification in mature seeds confirmed the biochemical consequences. OEmiR397/*OEmLAC14* seeds accumulated ~1.6-fold higher levels of soluble flavan-3-ol monomers (epicatechin and catechin) compared to *OEmLAC14* seeds, while insoluble polymeric CT content was reduced by ~40% (Figure 6F–I), demonstrating a direct impact on pathway output.

Collectively, heterologous expression in *Arabidopsis*, metabolite profiling, and molecular validation demonstrate that miR397-5p negatively influences seed coat CT biosynthesis by directly targeting and suppressing *SbLAC14*.

## 3. Discussion

Our study suggests a post-transcriptional regulatory node in sorghum seed metabolism by providing evidence that SbmiR397 directly targets SbLAC14 to negatively regulate the biosynthesis of condensed tannins (CTs) or proanthocyanidins (PAs) [22]. This finding, supported by molecular and transgenic approaches, suggests that the SbmiR397-SbLAC14 axis may serve as a potential modulator of phenolic flux. While laccases are implicated in various oxidative processes, their specific genetic regulation and role in PA polymerization in cereals have remained notably elusive [23,24]. Our data provide evidence consistent with the notion that SbLAC14 may function as a promiscuous oxidoreductase involved in the polymerization of flavan-3-ols in vivo, a conclusion supported by the reciprocal changes in polymer and monomer pools observed in our transgenic lines. This may place SbLAC14 at a metabolic branch point, potentially competing with or channeling substrates away from pathways leading to monomeric flavonoids and towards polymeric CT accumulation [25].

A central mechanistic insight from our work may be the post-transcriptional fine-tuning of this terminal biosynthetic step by a microRNA. The conservation of miR397 targeting of laccases, primarily documented in the context of lignin biosynthesis and copper homeostasis, may be repurposed here for the regulation of a distinct phenolic pathway [21]. Our dual-luciferase assay supported this interaction in sorghum [26]. The phenotypic and metabolic consequences—lightened seed color and reduced CTs upon SbmiR397 overexpression versus the opposite phenotype in plants expressing a miR397-resistant SbLAC14—are consistent and suggestive. This suggests that during seed development, the dynamic expression of SbmiR397 may act as a rheostat, regulating the abundance of SbLAC14 protein to potentially control the ultimate extent of flavan-3-ol polymerization [27]. This regulatory mechanism may allow the plant to rapidly adjust CT accumulation in response to developmental or environmental cues without altering the transcription of the core flavonoid structural genes, potentially offering a layer of metabolic plasticity.

Our results, however, raise several important questions that contextualize the significance and limitations of our findings. First, the biochemical specificity of SbLAC14 would benefit from further interrogation. Does it exhibit a true preference for flavan-3-ols like catechin over canonical monolignol substrates, or is its role in PA polymerization a function of its spatiotemporal co-expression with CT precursors in the seed coat vacuole [28]. The observed decrease in catechin and epicatechin levels upon SbLAC14 overexpression is suggestive of its in vivo role, but in vitro enzymatic assays with purified proteins are needed to define its kinetic parameters. Second, the upstream signals governing SbmiR397 transcription in the seed are completely unknown. Identifying the transcription factors that regulate this microRNA will connect this module to broader developmental programs or stress-signaling networks known to influence PA biosynthesis [29]. Third, we must consider functional redundancy. The sorghum genome encodes multiple laccase genes; the extent to which other *SbLACs* contribute to PA polymerization and whether they are similarly regulated by miR397 or other miRNAs remain open questions. The lack of a complete null mutant phenotype in our OEmiR397 lines may hint at such compensatory mechanisms. Fourth, in our study, SbLAC14–GFP fluorescence was examined in rice protoplasts, which suggests membrane association, but further co-localization studies in intact tissues are needed to define its exact subcellular compartment. Finally, while we observed differential *SbLAC14* expression between the representative lines 407B and 5833B, a quantitative correlation between *SbLAC14* transcript levels and CT content across the full panel of 32 sorghum accessions remains to be determined. Expanding expression analysis to a broader germplasm set would help establish whether this relationship holds across diverse genetic backgrounds.

Beyond the immediate biochemical pathway, the SbmiR397-SbLAC14 module may have tangible implications for sorghum trait engineering. The clear inverse relationship between SbmiR397 activity and CT content suggests a potential molecular strategy for manipulating this key nutritional and defensive metabolite [30]. For forage and feed applications where low tannin is desirable to enhance protein digestibility, overexpressing SbmiR397 or using gene editing to disrupt the SbLAC14 function may present a targeted approach [31]. Conversely, for developing sorghum varieties with enhanced antioxidant capacity for human consumption, strategies to dampen miR397 activity or to express a stabilized SbLAC14 mRNA in the seed could be employed. It is crucial, however, to investigate potential pleiotropic effects. Given the canonical role of laccases in lignification, altering this regulatory module might inadvertently affect stem strength or vascular development [23]. Our preliminary data on vegetative growth in transgenics showed no overt morphological defects, but a detailed analysis of stem biomechanics and lignin composition is necessary for a complete agronomic assessment.

## 4. Materials and Methods

### 4.1. Plant Materials

Thirty-two sorghum accessions used in this study were preserved by the Institute of Millet Crops, Hebei Academy of Agriculture and Forestry Sciences. All materials were planted at the Experimental Station of the Institute of Millet Crops, Hebei Academy of Agriculture and Forestry Sciences, Shijiazhuang, Hebei Province, China (114.6258° E, 37.9763° N) under the natural field conditions [32]. Based on the preliminary screening of condensed tannin (CT) content, two sorghum genotypes exhibiting contrasting levels of CT accumulation, 407B (low tannin) and 5833B (high tannin), were selected for dynamic analysis during seed development. Developing grains were sampled at eight developmental stages covering early grain formation through late maturation: 5 days post-anthesis (S1), 10 days post-anthesis (S2), 15 days post-anthesis (S3), 20 days post-anthesis (S4), 25 days post-anthesis (S5), 30 days post-anthesis (S6), 35 days post-anthesis (S7), 40 days post-anthesis (S8). All samples were collected with three independent biological replicates for subsequent analysis.

### 4.2. Measurement of Condensed Tannin Levels

Condensed tannin (CT) content in sorghum grains was determined using a commercial assay kit (Cat. No. HK0941; HengYi Biotechnology Co., Ltd., Yangzhou, China) based on the vanillin–HCl colorimetric method. In this assay, condensed tannins react with vanillin under acidic conditions to produce a red-colored product with maximum absorbance at 500 nm, and the color intensity is proportional to the CT concentration. Briefly, 0.05 g of freeze-dried and ground seed powder (passed through a 40-mesh sieve) was extracted with 0.5 mL of reagent I (extraction buffer) by ultrasonication for 30 min. After centrifugation at 12,000 rpm for 10 min at room temperature, the supernatant was collected as the sample extract. For the assay, 20 μL of sample extract, standard (100 μg/mL CT), or blank (reagent I) was mixed with 120 μL of reagent II (vanillin substrate) and 60 μL of HCl in a 96-well plate. The mixture was vortexed for 5 s, incubated at room temperature in the dark for 15 min, and the absorbance was measured at 500 nm using a microplate reader. The CT content was calculated based on the standard curve and expressed as μg per gram of dry weight (μg/g DW). All measurements were performed with three biological replicates per sample.

### 4.3. RNA-Seq Data Analysis and qRT-PCR Validation

Total RNA was extracted from seed samples using Trizol reagent (Invitrogen, Shanghai, China) following the manufacturer’s instructions. RNA-seq libraries were constructed and sequenced by MetWare (Wuhan, China) [33] on an Illumina NovaSeq 6000 (Illumina, San Diego, CA, USA) platform using a paired-end 150 bp (PE150) strategy, generating approximately 50 million raw reads per sample. Raw reads were quality-trimmed using Trimmomatic (v0.39) and then aligned to the sorghum reference genome (Sorghum bicolor v3.1.1, Phytozome) using HISAT2 (v2.2.1). The average mapping rate was 94%. Gene-level read counts were quantified using featureCounts (v2.0.3) against the annotation file of the same genome version. Differential expression analysis was performed with DESeq2 (v1.22.1) using default parameters; genes with an adjusted *p*-value (FDR) ≤ 0.05 and |log_2_(fold change)| ≥ 1 were defined as differentially expressed genes (DEGs). Transcript abundance was also calculated as FPKM (fragments per kilobase of transcript per million mapped reads) for visualization purposes. Quantitative real-time PCR (qRT-PCR) validation was conducted on the identical sample set. RNA isolation, cDNA synthesis, and qRT-PCR reactions followed the protocol established by Shi et al. [34], with three biological replicates employed. Primers targeting specific genes were designed via the NCBI Primer-BLAST tool (https://ncbi.nlm.nih.gov/tools/primer-blast/index.cgi, accessed on 18 January 2024).

### 4.4. Metabolome Sequencing

Metabolome analysis of sorghum grains (lines 407B and 5833B) at four developmental stages (S1–S4 post-anthesis) was performed by UPLC-MS/MSwith six biological replicates per stage (each pooled from ≥3 plants). Frozen samples were freeze-dried and ground (MM 400, Retsch GmbH, Haan, Germany; 30 Hz, 1.5 min) into fine powder for subsequent analyses, and then 50 mg powder was extracted with 1.2 mL 70% methanol containing internal standards. After vortexing (six cycles) and centrifugation (12,000 rpm, 10 min, 4 °C), supernatants were filtered (0.22 μm) for analysis. Chromatographic separation was performed on a SHIMADZU Nexera X2 UPLC (Shimadzu Corporation, Kyoto, Japan) system equipped with an ACQUITY BEH C18 column (2.1 × 100 mm, 1.7 μm; Waters Corporation, Milford, MA, USA) maintained at 40 °C. The mobile phase consisted of water (solvent A) and acetonitrile (solvent B), each containing 0.1% formic acid, delivered at a flow rate of 0.35 mL/min under gradient conditions. A triple quadrupole-linear ion trap mass spectrometer (SCIEX, Marlborough, MA, USA) equipped with an ESI source was used for MS analysis (operating at 550 °C; ion spray voltages of 5500 V in positive mode and −4500 V in negative mode). Metabolite identification and quantification used multiple reaction monitoring based on MetWare database and public libraries. Peak alignment and area extraction of the data were carried out using Analyst 1.6.3 software. Metabolic differences were assessed by PCA and OPLS-DA. Differentially accumulated metabolites (DAMs) were identified from the metabolomic profiling between 407B and 5833B across the four developmental stages (VIP ≥ 1, |log_2_FC| ≥ 1, *p* < 0.05).

### 4.5. Weighted Gene Co-Expression Network Analysis (WGCNA)

To identify co-expression modules associated with CT accumulation, we performed WGCNA using the WGCNA R package (v1.69). The analysis was conducted on all DEGs across the eight samples (407B and 5833B at S1–S4). After data filtering, the variance-stabilizing transformation was applied, and the signed network was constructed using the blockwiseModules function with the following parameters: soft-thresholding power β = 8 (selected based on the scale-free topology criterion R^2^ > 0.85), minModuleSize = 30, mergeCutHeight = 0.25, and deepSplit = 2. Module eigengenes (MEs) were calculated as the first principal component of each module. To identify trait-associated modules, we correlated MEs with CT content measured in the same samples (Figure 1C), and modules with |correlation| ≥ 0.7 and *p* < 0.05 were considered significantly associated with CT accumulation. Within each trait-associated module, hub genes were identified based on module membership (MM) and gene significance (GS), with thresholds of MM > 0.8 and GS > 0.8. The hub genes were further prioritized by their intramodular connectivity (kME).

### 4.6. Generation of Expression Constructs and Arabidopsis Genetic Transformation

Full-length SbLAC14 fragments were PCR-amplified from sorghum. Synthesized mSbLAC14 and SbmiR397-5p (Sangon, Shanghai, China) were cloned into the pRok2 vector. After transformation into Agrobacterium tumefaciens GV3101, the constructs were introduced into Arabidopsis thaliana (Col-0) and tt10 mutants using the floral dip method [35]. Transformants were selected on MS medium containing 25 mg/L kanamycin and grown to maturity. All transgenic lines used for phenotypic analysis were T3 homozygous, as confirmed by segregation analysis on selective medium, and at least three independent lines per construct were analyzed to minimize positional effects.

### 4.7. Quantitative Analysis of Flavonoids

UPLC-MS/MS analysis of flavonoid compounds including flavan-3-ol monomers and condensed tannins was performed as previously described [19]. Mature *Arabidopsis* seeds (0.05 g) were ground and extracted with 1 mL of 80% methanol at 4 °C for 24 h under constant shaking. After centrifugation (12,000 rpm, 15 min), the supernatant containing soluble flavan-3-ols was collected. The pellet was washed twice with 80% methanol, dried, and then re-extracted using 1 mL of 70% acetone supplemented with 0.1% formic acid at 4 °C over a 24 h period to retrieve the insoluble polymeric condensed tannins. Following centrifugation, the supernatant was dried, and the residue was dissolved in 1 mL of 80% methanol. Quantification was performed using standard curves established with catechin and epicatechin. Each sample was assessed using three biological replicates and three technical replicates.

### 4.8. DMACA Staining Method

DMACA staining was performed to visualize condensed tannins (CTs) in developing seeds [36]. Seeds dissected from siliques of wild-type *Arabidopsis*, *tt10* mutant, and *tt10*/35S::SbLAC14 lines were immersed in freshly prepared staining solution (1% *w*/*v* dimethylaminocinnamaldehyde in ethanol:6 M HCl = 1:1, *v*/*v*) for 15–30 min at room temperature in the dark. Following a quick rinse with distilled water, seeds were photographed under a stereomicroscope, where CTs were identified by their bright blue coloration.

### 4.9. 5′ Rapid Amplification of cDNA Ends (RACE)

Following the SMARTer RACE 5′/3′ Kit protocol (Clontech, Takara Bio Inc., Kusatsu, Shiga, Japan), 5′ RACE was conducted using 1 μg of total RNA extracted from tender leaves. After reverse transcription with a 5′ terminal adapter, PCR amplification was performed using an SbLAC14-specific primer. The amplified fragment was subsequently cloned into a vector for sequencing.

### 4.10. Dual-Luciferase Reporter (Dual-LUC) System Assay

For the dual-luciferase assay, the reporter vector was constructed by cloning the SbLAC14 ORF sequence (containing the putative miR397-5p binding site) downstream of the firefly luciferase (LUC) gene in the pGreenII-0800-LUC vector. The effector vector was constructed by cloning the SbmiR397-5p precursor into the pGreenII-62-SK overexpression vector, driven by the CaMV 35S promoter. The recombinant reporter and effector vectors were separately introduced into *Agrobacterium tumefaciens* strain GV3101. Following co-infiltration into *Nicotiana benthamiana* leaves, dual-luciferase activity was detected by spraying D-luciferin and imaging with an In Vivo Plant Imaging System (Berthold Technologies, GmbH & Co. KG, Bad Wildbad, Germany, Germany). The empty vectors pGreenII-0800-LUC and pGreenII-62-SK were used as negative controls.

### 4.11. Subcellular Localization Analysis

To determine subcellular localization of SbLAC14, an SbLAC14-GFP fusion was expressed under the CaMV 35S promoter. The SbLAC14 CDS was PCR-amplified with primers (5′-GGATCCACCGGGTAGTTTAGGCACTG-3′ and 5′-TCTAGACATCCATGGCTCATGCCTTTG-3′) to introduce a BamHI site at the 5′ end and an XbaI site at the 3′ end. The amplified fragment was cloned into pMD18-T and sequenced. The wild-type SbLAC14 was then digested and in-frame fused to the 5′ end of GFP. The resulting construct was transformed into rice protoplasts, which were mounted on glass slides and observed under a Zeiss LSM700 confocal microscope (Carl Zeiss, Inc., Thornwood, NY, USA).

### 4.12. Statistical Analysis

Statistical computations were performed with GraphPad Prism 8 (GraphPad Software, San Diego, CA, USA). One-way ANOVA or Student’s *t*-test was applied to assess statistical significance, with *p* ≤ 0.05 considered significant. All data are presented as mean values derived from at least three biological replicates.

## 5. Conclusions

In conclusion, the present work suggests that *SbLAC14* may function as a laccase gene preferentially expressed in the sorghum seed coat and may contribute to the polymerization of flavan-3-ol units into condensed tannins (CTs), potentially influencing seed coat pigmentation. Our data also indicate that miR397-5p likely targets and cleaves SbLAC14 mRNA, pointing to a possible regulatory interaction between them in tannin accumulation. Furthermore, our results suggest that CT accumulation is a dynamic, stage-specific process occurring mainly during early to mid-seed development, with coordinated transcriptomic reprogramming. Collectively, the miR397-5p–SbLAC14 node emerges as a candidate regulatory axis that may offer genetic targets for future sorghum breeding aimed at modulating tannin content for improved nutritional and industrial qualities.

## Data Availability

The data supporting this study are available from the corresponding author upon reasonable request. The RNA-seq data have been deposited in the NCBI Sequence Read Archive (SRA) under BioProject accession number PRJNA1429536.

## Supplementary Materials

The following supporting information can be downloaded at https://www.mdpi.com/article/10.3390/plants15152339/s1. Figure S1: Comparison of seed coat structures between two samples. Figure S2: Correlation analysis between RNA-seq and RT-qPCR results for selected genes. Figure S3: Expression profiles of SbLAC gene family members across various sorghum tissues. Figure S4: Molecular characterization and additional phenotypic analyses of SbLAC14 transgenic *Arabidopsis* lines. Figure S5: Design of miR397-5p-resistant SbLAC14 construct and expression analysis in transgenic *Arabidopsis* lines. Table S1: Significant DEGs of S1–S4 stage between 407B and 5833B. Table S2: Genes in 9 clusters. Table S3: Gene relative expression of anthocyanin and condensed tannin biosynthesis. Table S4: Significant DAMs of S1–S4 stage in 407B and 5833B. Table S5: Summary of *SbLAC* gene family.
