# Supplementary material for: Integrated Transcriptomic and Metabolomic Profiling Reveals the Involvement of the miR397-5p–SbLAC14 Module in Condensed Tannin Accumulation in Developing Sorghum Seeds
Source: Plants (Basel). 2026 Jul 29;15(15):2339. doi: 10.3390/plants15152339 (PMC13468490; doi:10.3390/plants15152339)
Supplement: Supplementary file 1 [file plants-15-02339-s001.zip › Supplementary figures.pdf]

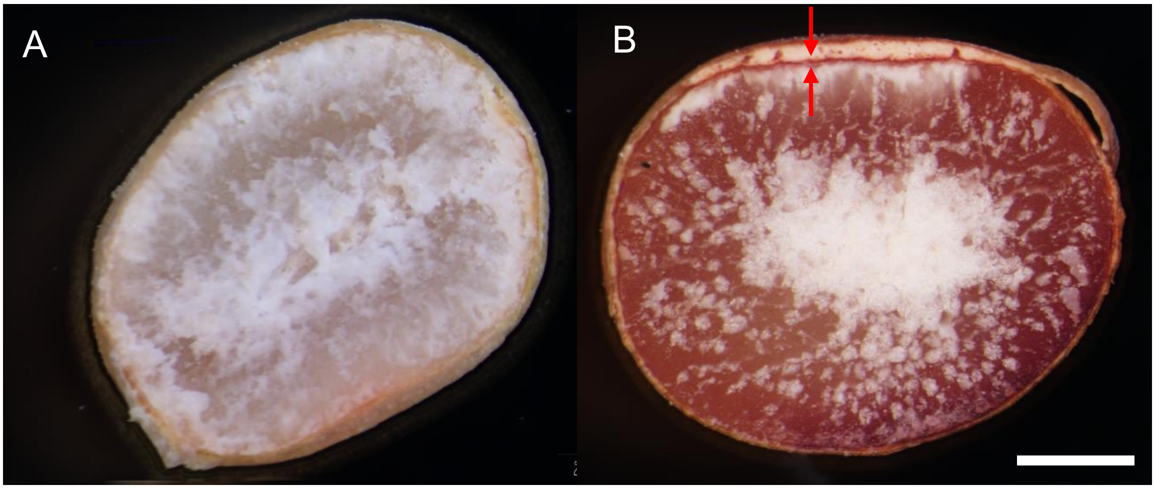

Fig. S1. Comparison of seed coat structures between two samples at S5 stage. (A) Sample 5833B, showing the presence of a distinct tannin layer. (B) Sample 407B, lacking a tannin layer. Both images are light micrographs of cross-sections (indicated by red arrows). Scale bars: 2 mm.

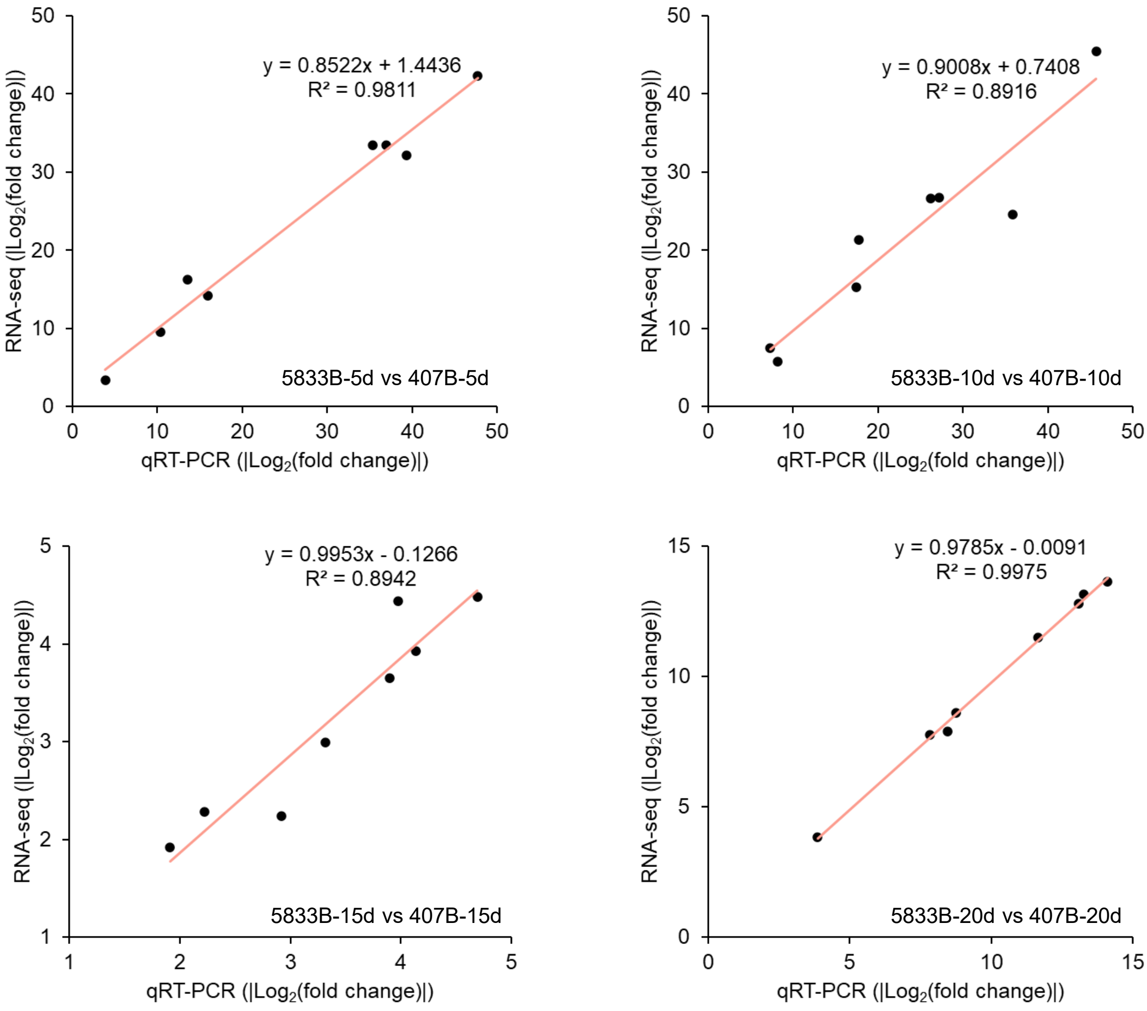

Fig. S2. Correlation analysis between RNA-seq and RT-qPCR results for selected genes. Each point represents an individual gene (n = 8). Values are $\log_2$-transformed fold changes (5833B vs 407B) from qRT-PCR (x-axis) and RNA-seq (y-axis). The linear regression line is shown in red.

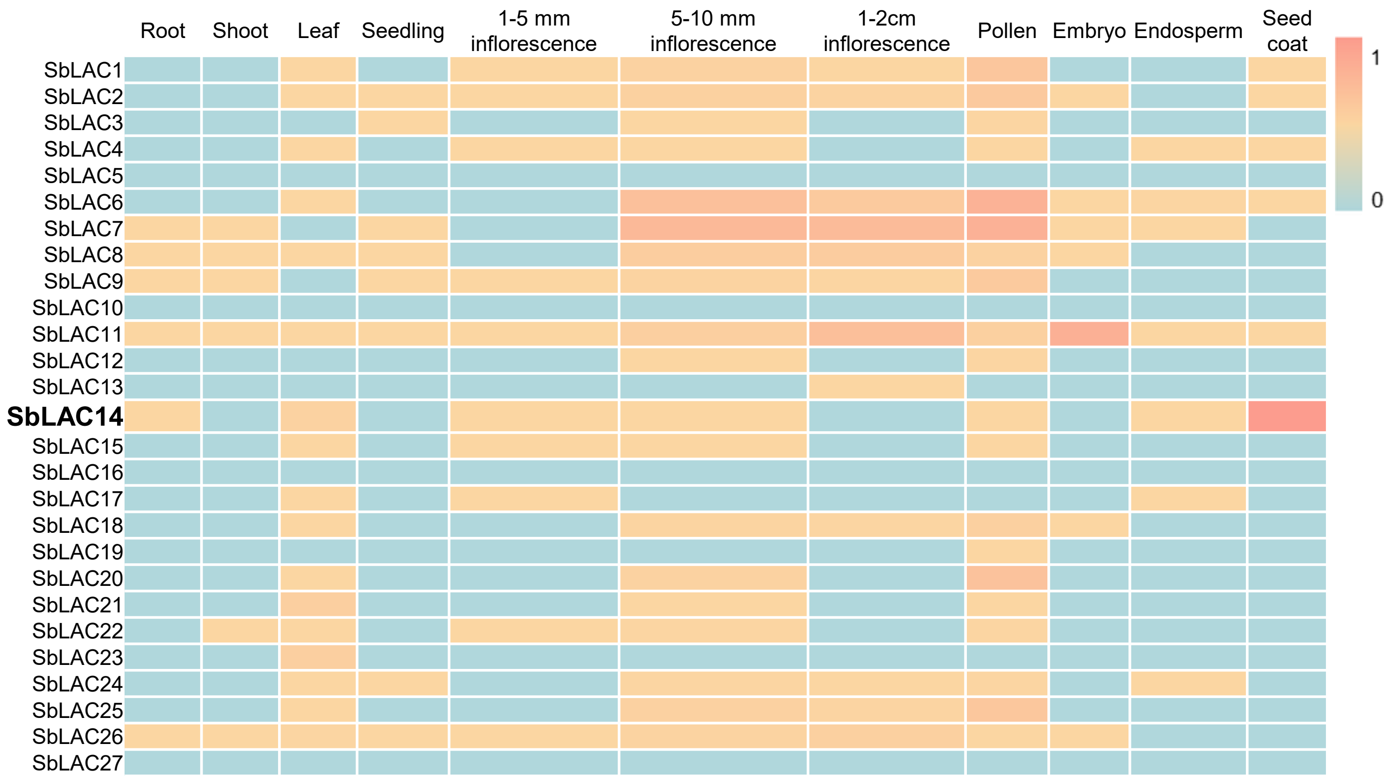

Fig. S3. Expression profiles of *SbLAC* gene family members across various sorghum tissues. Heatmap showing transcript levels of *SbLAC* genes in 11 different tissues/organs, including root, shoot, leaf, seedling, inflorescence at three developmental stages (1–5 mm, 5–10 mm, and 1–2 cm), pollen, embryo, endosperm, and seed coat. Expression values were log_2-transformed and normalized per gene (scale 0–1). Color scale from blue (low expression) to red (high expression) represents relative transcript abundance. *SbLAC14* is highlighted in the heatmap, showing its predominant expression in seed coat and reproductive tissues.

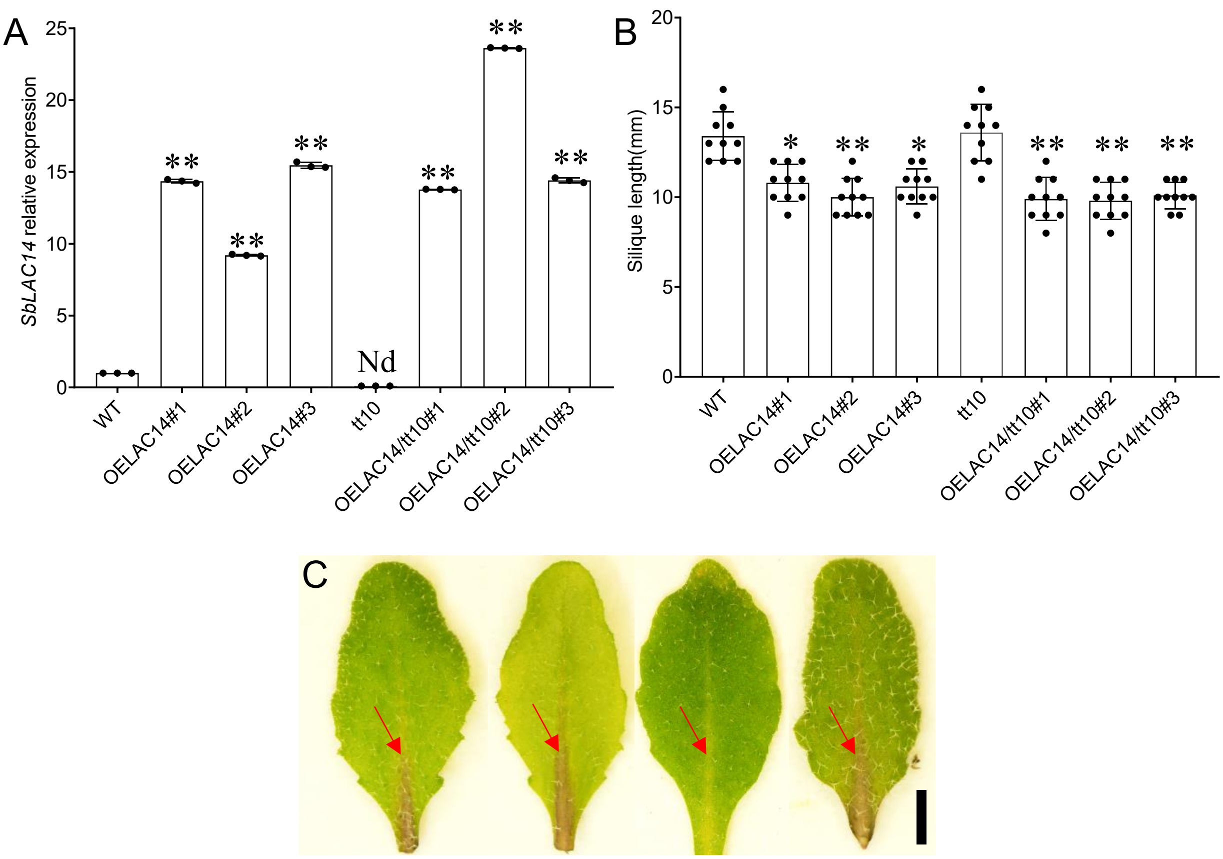

Fig. S4. Molecular characterization and additional phenotypic analyses of SbLAC14 transgenic Arabidopsis lines.

(A) SbLAC14 transcript abundance relative to controls across four Arabidopsis lines: wild-type (WT, Col-0), SbLAC14-overexpressing WT (OE-LAC14/WT), *tt10* mutant, and SbLAC14-overexpressing *tt10* mutant (OE-LAC14/*tt10*). Values are shown as mean ± SD from three biological replicates (n=3). Nd, not detected.

(B) Silique length measurements in the four Arabidopsis lines. At least 10 siliques per line were measured. Values are mean ± SD from three biological replicates (n=3). Asterisks denote significant differences relative to OE-LAC14/tt10 (* $p < 0.05$, ** $p < 0.01$; Student's t-test).

(C) Leaf phenotypes of the four Arabidopsis lines. Red arrows indicate the midrib color variation among genotypes, with pigmentation visible in WT, OE-LAC14/WT, and OE-LAC14/*tt10* lines. Scale bars: 2 mm.

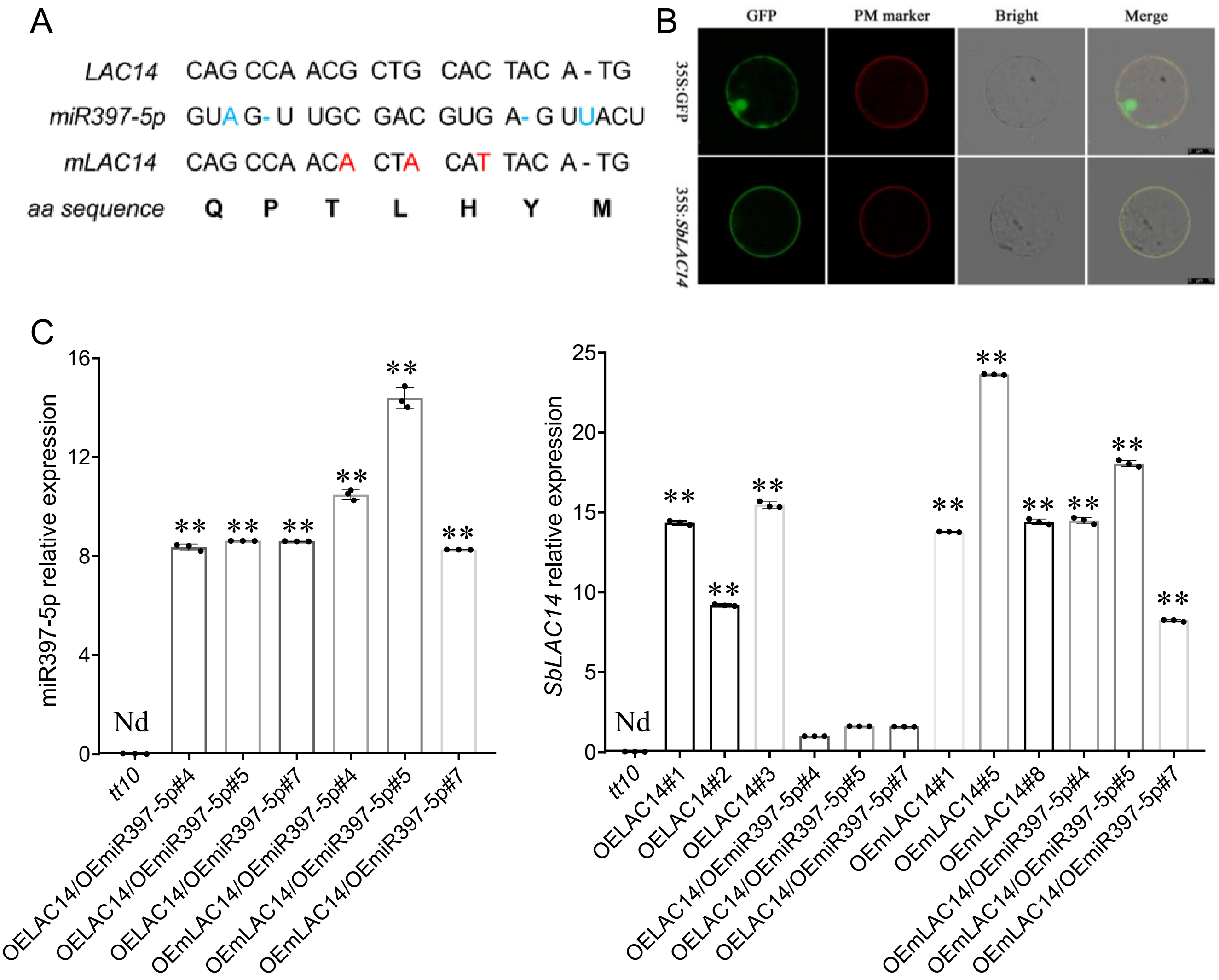

Fig. S5. Design of miR397-5p-resistant SbLAC14 construct and expression analysis in transgenic Arabidopsis lines.

(A) Schematic diagram showing the construction of miR397-5p-resistant SbLAC14 (mLAC14). Upper panel: alignment of miR397-5p sequence with wild-type LAC14 and mutated LAC14 (mLAC14) mRNA sequences. Non-complementary nucleotides between miR397-5p and the native LAC14 sequence are marked in blue. Engineered single-base substitutions within the miR397-5p target region of LAC14 are indicated in red. These silent mutations disrupt miRNA complementarity without altering the amino acid sequence.

(B) Cellular distribution of the SbLAC14-GFP fusion protein expressed in rice protoplasts. Chlorophyll autofluorescence (red), GFP fluorescence (green), DIC image, and merged image are shown. Scale bars = 10 μm.

(C) Relative expression levels of miR397-5p and SbLAC14 in five Arabidopsis lines: tt10 mutant, SbLAC14-overexpressing tt10 (OE-LAC14/tt10), OE-LAC14/OE-miR397-5p in tt10 background, mutated SbLAC14-overexpressing tt10 (OE-mLAC14/tt10), and OE-mLAC14/OE-miR397-5p in tt10 background. qRT-PCR was used to quantify transcript levels, which were then normalized against an endogenous control gene. Nd, not detected. Values are mean ± SD from three biological replicates (n=3). Asterisks denote significant differences relative to OE-LAC14/tt10 (* $p < 0.05$, ** $p < 0.01$; Student's t-test).
